# Respiratory effects of off label anakinra in critically ill neonates: a 15-patient observational cohort study

**DOI:** 10.3389/fped.2026.1806329

**Published:** 2026-05-15

**Authors:** Domenico Umberto De Rose, Gaia Maria Cesaroni, Francesca Campi, Chiara Maddaloni, Sara Ronci, Stefano Caoci, Irma Capolupo, Andrea Dotta

**Affiliations:** Neonatal Intensive Care Unit, “Bambino Gesù” Children's Hospital IRCCS, Rome, Italy

**Keywords:** anakinra, bronchopulmonary dysplasia, IL-1, infant, newborn, respiratory distress syndrome

## Abstract

**Background:**

Interleukin-1 (IL-1)–mediated inflammation can contribute to lung injury, impaired alveolar development, and pulmonary vascular dysfunction in critically ill neonates. Anakinra, a recombinant IL-1 receptor antagonist, has emerged as a potential adjunct therapy in severe neonatal hyperinflammatory and respiratory conditions, but evidence in this population remains limited.

**Methods:**

We conducted a retrospective observational study of 15 neonates treated off label with anakinra (10 mg/kg/day in two doses) at a tertiary neonatal intensive care unit between 2021 and 2025. Clinical indications included severe or evolving bronchopulmonary dysplasia (BPD), secondary hemophagocytic lymphohistiocytosis (HLH), pulmonary hypertension, interstitial lung disease and complex congenital anomalies. Respiratory support was evaluated at treatment initiation and at predefined follow-up timepoints up to 60 days.

**Results:**

At baseline, 73% of infants required mechanical ventilation and 27% non-invasive ventilation. By the end of treatment (data available for 14/15 infants), mechanical ventilation decreased to 21% (absolute reduction 52%, relative reduction 71%), while spontaneous breathing with or without supplemental oxygen increased to 64%. All infants survived. Anakinra was generally well tolerated: transient transaminase elevations occurred in 40%, γGT increases in 20%, and transient neutropenia in 13%, with no clinically significant hepatotoxicity, allergic reactions, or seizures.

**Conclusions:**

In this largest neonatal cohort to date, Anakinra administration was associated with improvements in respiratory status, although a direct causal relationship cannot be established, and a favorable safety profile. Multicenter randomized controlled trials are warranted to confirm these findings, especially in preterm infants at risk of developing bronchopulmonary dysplasia.

## Introduction

Interleukin-1 (IL-1) is a central mediator of sterile and infection-related inflammation, and its dysregulation has been implicated in several inflammatory and cardiorespiratory conditions (1, 2). Anakinra, a recombinant IL-1 receptor antagonist (IL-1Ra), is increasingly used off label in critically ill infants when conventional anti-inflammatory strategies fail to produce clinical improvement (3, 4). Evidence in the neonatal population, however, remains limited. The largest previously published neonatal experience includes small case series (such as our earlier report of five infants treated for refractory hyperinflammatory and pulmonary conditions) showing rapid reduction in inflammatory markers, good safety, and potential respiratory benefits, including improved lung compliance and facilitation of ventilatory weaning (5).

The Anakinra Pilot trial, in which extremely preterm infants receive intravenous anakinra to prevent bronchopulmonary dysplasia (BPD), starting within the first 24 h of life and continuing for 21 days, is still ongoing (6).

The specific respiratory effects of Anakinra have not yet been systematically explored in a larger real-world cohort. Severe bronchopulmonary dysplasia (BPD), pulmonary hypertension, interstitial lung disease, or systemic hyperinflammatory states may all converge into respiratory deterioration that is often unresponsive to steroids alone (7, 8). In these contexts, IL-1–mediated pathways have been proposed as contributors to lung injury, impaired alveolar development, and abnormal pulmonary vascular remodeling, providing a rationale for therapeutic IL-1 blockade (9, 10).

A recent study from Li et al. (11) examines longitudinal IL-1 cytokine profiles in extremely preterm infants and identifies elevated IL-1α at postnatal day 28 as an independent predictor of BPD.

In this study, we described 15 neonates treated off label with anakinra, focusing on its impact on respiratory support trajectories. Our primary aim was to evaluate changes in respiratory support over time and to assess safety in this fragile population.

## Methods

### Study design and setting

We conducted a retrospective observational cohort study at the Neonatal Intensive Care Unit (NICU) of the “Bambino Gesù” Children's Hospital IRCCS (Rome, Italy), evaluating all neonates and infants up to 3 months of age treated off label with anakinra between 2021 and 2025.

Patients were identified through a systematic review of medical records, and all consecutive patients who received at least one dose of anakinra during the study period were included. Therefore, the study cohort represents the entire population of treated patients in our center over the specified timeframe. A carefully chosen cohort of patients with severe, resistant diseases that were not improving with conventional treatments were given consideration for anakinra. Clinical indications included severe or evolving bronchopulmonary dysplasia (BPD), secondary hemophagocytic lymphohistiocytosis (HLH), pulmonary hypertension, interstitial lung disease and complex congenital anomalies. The overall number of patients in whom anakinra was considered but not administered was not methodically recorded because of the retrospective nature. All patients received concomitant standard-of-care therapies according to their underlying condition, including respiratory support (invasive or non-invasive ventilation), corticosteroids, diuretics or/and antimicrobial treatments.

### Patients and treatment protocol

All consecutive patients treated with off label anakinra were included. The decision to initiate off label anakinra was made within a multidisciplinary team, including neonatologists, pediatric rheumatologists and, when appropriate, oncohematology and infectious disease specialists. Treatment was administered following institutional procedures for use of off label therapies.

Anakinra was considered in patients with disease refractory to standard treatments, based on predefined criteria, including inadequate response to first-line therapies and persistent severe disease activity. The decision to treat was supported by available evidence from the literature and clinical experience. Patients were not selected based on a single underlying diagnosis, but rather on the presence of a shared hyperinflammatory phenotype, refractory to standard therapies, across heterogeneous clinical conditions.

The study was reviewed and approved by the Scientific Direction of “Bambino Gesù” Children's Hospital (code Maddaloni RAP-2025-0001) According to local regulations, formal Institutional Review Board/Ethics Committee approval was not required for this type of observational, retrospective analysis. Written informed consent was obtained from the parents or legal guardians prior to treatment initiation.

Patients were closely monitored during therapy for clinical response and potential adverse events, according to standard safety protocols in our institution.

Anakinra was administered at a dose of 10 mg/kg/day, divided into two daily doses, either intravenously or subcutaneously depending on the patient's clinical condition, as previously described (5).

### Timepoints and data collection

From electronic medical records, we extracted type and level of respiratory support (mechanical ventilation, non-invasive ventilation, spontaneous breathing with or without supplemental oxygen); timing of respiratory changes at standardized intervals: baseline (T0, start of anakinra), day 15 (T1), day 30 (T2), and day 60 (T3); inflammatory markers, microbiology, organ dysfunction, and imaging when available; adverse events (hepatic toxicity, cytopenias, allergic reactions, seizures); in-hospital mortality.

### Outcomes

The primary outcome was the evolution of respiratory support over time (mechanical ventilation, non-invasive ventilation, or spontaneous breathing with/without supplemental oxygen). Secondary outcomes included adverse events drug-related (transaminase elevation, γGT elevation, neutropenia, seizures, allergic reactions) and overall in-hospital mortality.

### Statistical analysis

All statistical analyses were performed using IBM SPSS Statistics 25. Continuous variables were summarized as median and interquartile range (IQR) due to the small sample size and non-normal distribution. Categorical variables were expressed as absolute numbers and percentages.

Because the respiratory support categories represent an ordinal severity scale, changes over time were evaluated using Wilcoxon signed-rank test for paired comparisons between baseline and final respiratory status. Absolute and relative reductions in mechanical ventilation were calculated as absolute risk difference and relative percentage reduction.

Longitudinal descriptive trajectories were plotted for each patient to visualize transitions in respiratory support (mechanical ventilation to NIV or to spontaneous breathing/supplemental oxygen); because of the limited sample size, no mixed-effects modeling was applied, but temporal patterns were only qualitatively compared.

One infant was transferred to another institution during treatment; therefore, end-of-therapy respiratory data were unavailable. This case was included in baseline and early time point analyses but excluded from final respiratory outcome calculations, consistent with standard practice for observational neonatal cohorts.

A two-sided *p* value < 0.05 was considered statistically significant. Given the small sample size and exploratory nature of the study, results were primarily interpreted for clinical relevance rather than strict hypothesis testing.

## Results

### Patients

Fifteen consecutive patients were studied, including 5 previously reported (5). Median gestational age at birth was 30 weeks (IQR 25–37). Ten patients (67%) were born preterm; 10/15 (67%) were males. Median weight at treatment start was 2450 g (IQR 1,400–3,186 g). Clinical indications for Anakinra included: severe or evolving BPD (*n* = 10), secondary Hemophagocytic Lymphohistiocytosis (HLH) following bacterial sepsis (*n* = 2), primary pulmonary hypertension due to genetic variants (*n* = 2), interstitial lung disease (*n* = 1), and recurrent polyserositis (*n* = 1) (Table 1).

**Table 1:** Clinical characteristics of the 15 neonates treated with anakinra.

Patient	Gestational age at birth (weeks)	Birthweight (grams)	Postmenstrual age at the start of Anakinra (weeks)	Respiratory support at the start of Anakinra (days)	Main diagnosis/indication for Anakinra	Prior failed therapies
1	34	1,550	43	SIPPV	Secondary HLH following *Klebsiella pneumoniae* infection; Bartter syndrome; late prematurity.	Postnatal steroids, Diuretics, Antibiotics
2	23	550	44	HFOV	Secondary HLH following *Enterobacter cloacae* infection; extreme prematurity; BPD; spontaneous intestinal perforation requiring ileostomy.	Postnatal steroids, Diuretics, Antibiotics
3	38	3,070	45	SIMV	Chronic lung disease; recurrent polyserositis; multiple congenital anomalies including complex congenital heart disease.	Postnatal steroids, Diuretics
4	37	2,840	42	nIPPV	Chronic lung disease; primary pulmonary hypertension (heterozygous missense mutation in *KCNK3*); single kidney.	Postnatal steroids, Diuretics
5	38	2,900	47	nIPPV	Interstitial lung disease; persistent pulmonary hypertension post-ECMO.	Postnatal steroids, Diuretics
6	25	690	44	SIPPV	BPD; extreme prematurity.	Postnatal steroids, Diuretics
7	24	570	33	nIPPV	BPD; extreme prematurity; severe post-hemorrhagic hydrocephalus requiring repeated shunt revisions.	Postnatal steroids, Diuretics
8	29	1,940	30	SIPPV	BPD; severe prematurity; hydrops; paroxysmal supraventricular tachycardia.	Postnatal steroids, Diuretics
9	30	2,000	33	HFOV	BPD; severe prematurity; cystic hygroma/hydrops; Noonan syndrome.	Postnatal steroids, Diuretics
10	38	3,720	41	SIPPV	Primary pulmonary hypertension (TBX4 mutation).	Postnatal steroids, Diuretics
11	30	750	41	SIPPV	BPD; severe prematurity; hydrocephalus.	Postnatal steroids, Diuretics
12	28	1,100	32	SIPPV	Chronic lung disease; Tetralogy of Fallot; severe prematurity.	Diuretics
13	37	3,640	39	SIPPV	Diaphragmatic relaxation following perinatal asphyxia requiring respiratory support; subcutaneous fat necrosis.	Postnatal steroids
14	30	900	33	HFOV	BPD; severe prematurity; ROP.	Postnatal steroids, Diuretics
15	25	600	33	SIPPV	BPD; extreme prematurity; subglottic stenosis; ROP.	Postnatal steroids, Diuretics

**Figure 1:**
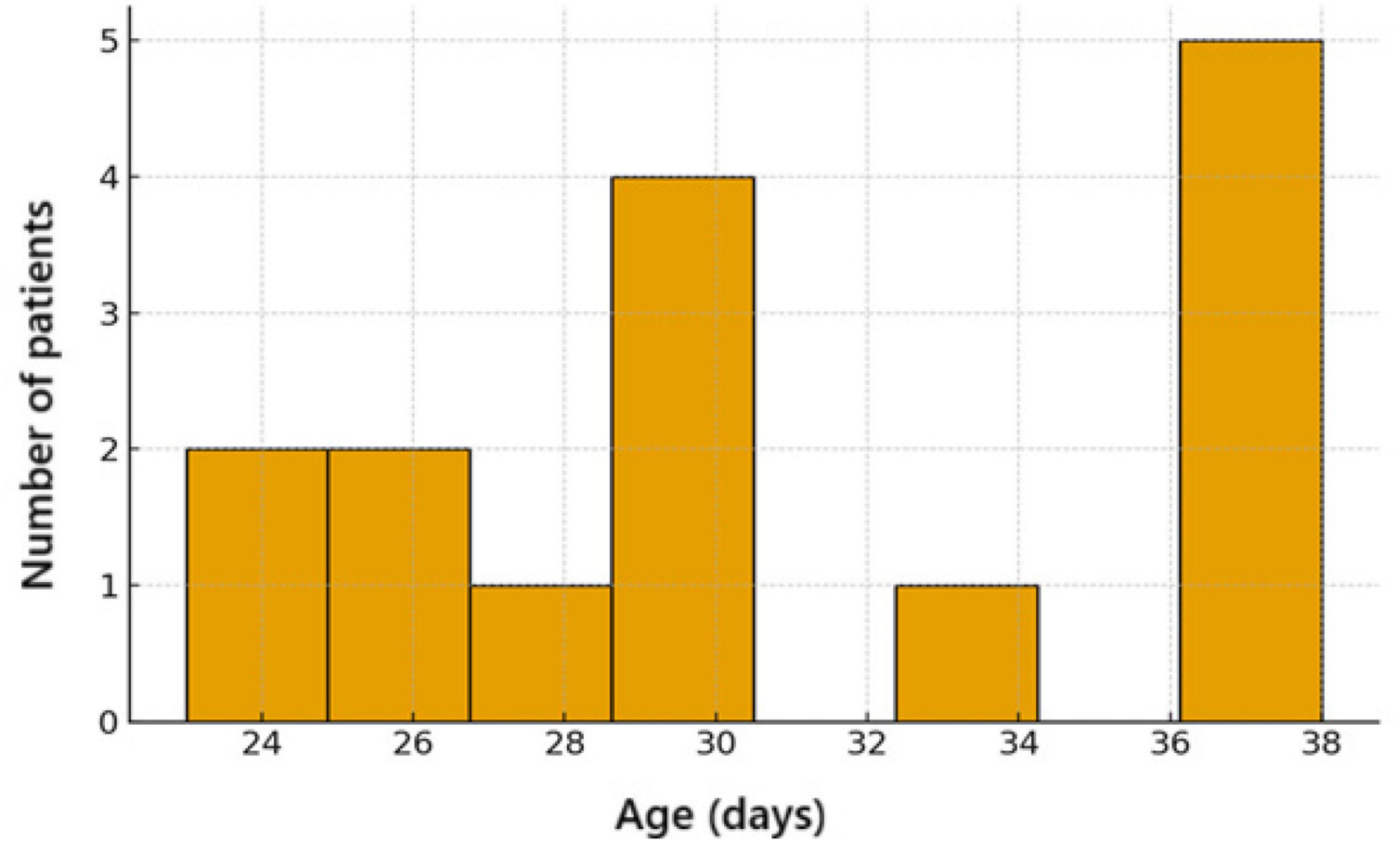
Chronological age distribution of included patients at the start of anakinra.

Anakinra was started at about one month of life (Figure 1).

### Baseline respiratory status

At start of Anakinra, all patients were receiving respiratory support, with 11/15 (73%) on invasive mechanical ventilation (MV) and 4/15 (27%) on non-invasive ventilation (NIV). The cohort showed a high severity: 80% (12/15) experienced at least one late-onset sepsis episode during NICU stay; several had pulmonary hypertension, interstitial lung disease, or complex congenital anomalies.

### Improvement in respiratory support

By the end of treatment (data available for 14 infants, because one infant was transferred to another hospital and respiratory data at the end of treatment were unavailable), patients on mechanical ventilation decreased from 73% to 21%, with an absolute reduction of 52% and a relative reduction of 71%. NIV decreased from 27% to 7%. Spontaneous breathing with or without supplemental oxygen increased from 0 to 64%.

**Figure 2:**
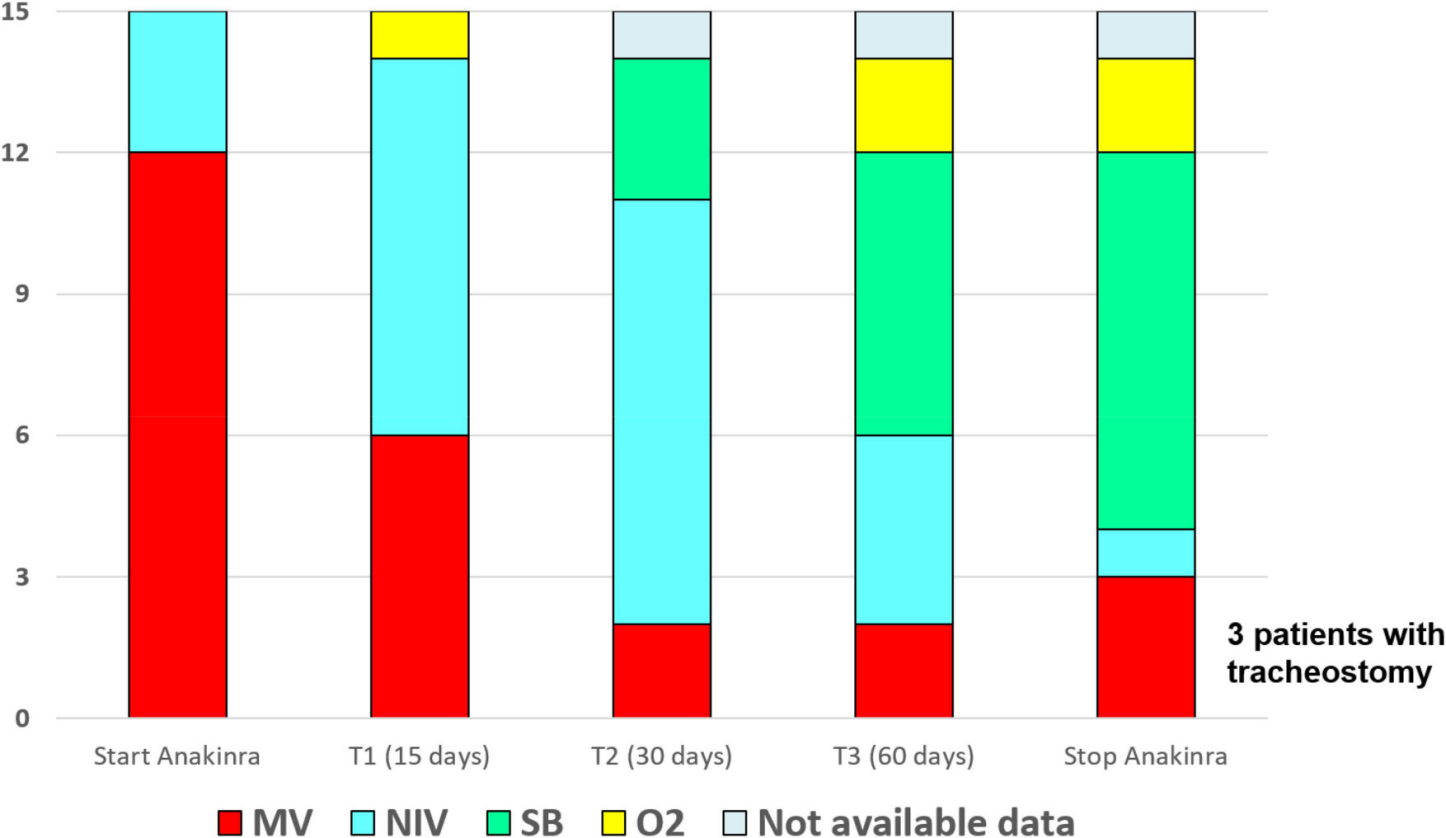
Evolution of respiratory support in 15 neonates treated with anakinra. Stacked bars represent the distribution of respiratory support modalities at five predefined time points: treatment initiation (Start Anakinra), day 15 (T1), day 30 (T2), day 60 (T3), and end of therapy (Stop Anakinra). Respiratory categories include mechanical ventilation (MV), non-invasive ventilation (NIV), spontaneous breathing (SB), supplemental oxygen (O_2_), and unavailable data. A progressive decline in MV and NIV was observed over time, with a corresponding increase in spontaneous breathing and oxygen-only support.

A graphical analysis of respiratory trajectories (Figure 2) showed a progressive improvement in respiratory status during anakinra therapy, starting within the first 15 days in most infants, and a stabilization or further gains by day 30 and day 60. In three infants requiring tracheostomy (TS), only partial respiratory benefit was noted.

### Safety data and in-hospital mortality

No clinically significant hepatotoxicity occurred (0/15), while transient hypertransaminasaemia occurred in 6/15 patients (40%). Transient Gamma-Glutamyl Transferase (γGT) elevation was evident in 3/15 patients (20%). Transient neutropenia was displayed in 2/15 patients (13%). No allergic reactions or seizures were reported. Retinopathy of prematurity (ROP) occurred in 4/10 preterm infants, all of whom had severe BPD and prolonged ventilation/oxygen requirements (that we interpreted as consistent with disease severity rather than treatment). All 15 infants survived.

## Discussion

In this paper, we describe the respiratory course of critically ill neonates who received off-label Anakinra. Within this cohort, we observed a reduction in the proportion of infants requiring invasive mechanical ventilation (from 73% to 21%) and an increase in spontaneous breathing with or without supplemental oxygen (from 0% to 64%) over time. These results imply that in a variety of neonatal diseases marked by severe lung injury, systemic inflammation, or both, IL-1 receptor antagonism may significantly improve respiratory function.

The increasing amount of preclinical evidence linking IL-1-mediated inflammation to vascular remodeling, altered alveolarization, and newborn lung damage provides a molecular explanation for our findings.

Blocking IL-1 signaling with an IL-1 receptor antagonist decreased inflammation-driven lung damage under mild hyperoxia in a mouse model of bronchopulmonary dysplasia, suggesting that IL-1α/β are key disease drivers (12). Another preclinical murine model of pulmonary hypertension linked to bronchopulmonary dysplasia was treated with daily administration of an IL-1 receptor antagonist, which preserved the pulmonary microvascular structure, prevented increases in pulmonary vascular resistance, and decreased cardiac fibrosis (13). These results suggest that IL-1Ra is a viable treatment option for BPD and BPD-PH.

Green et al. (2023) (9) highlight the role of the IL-1 family in complications of prematurity, emphasizing that IL-1β triggers downstream MyD88/NF-κB signalling that can impair lung development, and that IL-1Ra (anakinra) has shown protective effects in animal models of BPD and BPD-associated pulmonary hypertension.

Higher circulating IL-1α levels at postnatal day 28 were independently associated with the development of bronchopulmonary dysplasia in extremely preterm infants, highlighting a potential biomarker of disease risk (11).

From the perspective of clinical translation, the review by Durlak lists new treatments for BPD, such as IL-1R antagonists, and emphasizes that anakinra is one of the most physiologically feasible novel interventions, despite the lack of efficacy data (14).

Together, these data provide a solid theoretical basis for our observation of improved respiratory trajectories, despite the complex comorbidities of our cohort.

Interruption of IL-1-driven inflammatory cascades in the lung may be the cause of our patients' early respiratory improvement, with many demonstrating improvement within the first 15 days of treatment. Anakinra may reduce lung inflammation more quickly, encourage alveolar and vascular regeneration, and enable quicker ventilator weaning by blocking IL-1 signaling.

The choice of anakinra dosage (10 mg/kg/day divided into two doses) was based on previous paediatric experience in hyperinflammatory conditions, where high weight-based doses are often required to ensure effective interleukin-1 blockade (15–17).

Given the lack of standardized dosing regimens in neonates, patients were closely monitored. The safety profile matched our findings from the previous case series (5). Importantly, all patients survived, and we observed only transient and manageable laboratory abnormalities and no clinically significant adverse effects. The absence of clinically significant hepatotoxicity, allergic reactions or seizures in our cohort further supports the tolerability of this therapeutic approach in a fragile neonatal population.

This study has several limitations. Firstly, the retrospective design, with the absence of randomization and standardized treatment protocols that limit the ability to infer causality between anakinra and respiratory improvement. Secondly, we did not have a control group and most infants received concomitant postnatal steroids (93%). Thus, the degree to which anakinra independently contributed to respiratory improvement cannot be isolated. As a result, the observed clinical improvement should be regarded with caution, as it could be also due to the natural course of the disease or the action of concomitant standard therapy.

Additionally, a potential selection bias was introduced because the patients were chosen based on clinical judgment in the context of severe and refractory disease and we did not consider a denominator population. The study cohort may not be representative of the larger neonatal community with comparable conditions because anakinra was given preferentially to a well selected subgroup of severely ill neonates.

Indeed, this cohort was heterogeneous and indications for Anakinra ranged from HLH to severe BPD, pulmonary hypertension, and interstitial lung disease. Disease trajectories may differ substantially, limiting generalizability. This heterogeneity further complicates the interpretation of treatment effects, as the response to IL-1 blockade may vary depending on the underlying disease mechanism and clinical context.

Although the stepwise improvement in respiratory support was clear, detailed ventilatory parameters (e.g., MAP, PIP, FiO_2_, compliance indices) were not collected and analyzed systematically at all time points, because of the small sample size and the heterogeneity in respiratory strategies at the different timepoints.

Despite being the largest neonatal cohort published to date, the sample remains limited and these findings support the hypothesis (suggested in the earlier 5-patient case series) that IL-1 inhibition may contribute to improved lung stability and facilitate ventilatory weaning in select critically ill neonates. However, given the single-center methodology and the specific clinical environment, caution should be exercised when applying these findings to other institutions or patient populations.

Our findings correspond with accumulating evidence that IL-1 plays a critical role in promoting both systemic and brain inflammation during the perinatal period. By blocking IL-1 signaling, anakinra not only mitigates peripheral inflammation but also shows promise as a treatment technique to preserve the developing brain and lower the risk of long-term neurodevelopmental abnormalities in the fragile preterm population (18).

Given the favourable safety profile and observed improvement in respiratory support demands, our findings urge the design of a prospective, multicenter studies with more homogeneous patient populations and standardized treatment protocols to better define the therapeutic role of anakinra in neonatal inflammatory and respiratory conditions, and to identify the subgroups most likely to benefit from IL-1–targeted interventions.

## Conclusions

In this largest neonatal cohort reported to date, Anakinra was associated with a marked improvement in respiratory outcomes across a heterogeneous group of critically ill neonates with severe comorbidities. A progressive reduction in the need for mechanical ventilation and non-invasive respiratory support was observed throughout treatment, with the majority of infants transitioning to spontaneous breathing by the end of therapy. Although causality cannot be definitively established in the absence of a control group and given the complexity of underlying conditions, the consistency of respiratory improvement across diverse pathologies supports our hypothesis that targeting IL-1–mediated pathways may mitigate pulmonary inflammation and stabilize lung function. Our findings suggest that in neonates with severe respiratory failure, evolving BPD, pulmonary hypertension or interstitial lung disease, especially when conventional therapies are insufficient, Anakinra may represent a viable adjunctive therapy. However, prospective, multicenter randomized controlled trials with standardized respiratory assessments are needed to confirm these results or not.

## Data Availability

The datasets presented in this article are not readily available because the dataset is not accessible. Requests to access the datasets should be directed to chiara.maddaloni@gmail.com.

## References

[1] Dinarello CA Simon A Van Der Meer JWM. Treating inflammation by blocking interleukin-1 in a broad spectrum of diseases. Nat Rev Drug Discov. (2012) 11(8):633–52. 10.1038/nrd3800 22850787 PMC3644509

[2] Sota J Vitale A Insalaco A Sfriso P Lopalco G Emmi G Safety profile of the interleukin-1 inhibitors anakinra and canakinumab in real-life clinical practice: a nationwide multicenter retrospective observational study. Clin Rheumatol. (2018) 37(8):2233–40. 10.1007/s10067-018-4119-x 29770930

[3] Maniscalco V Abu-Rumeileh S Mastrolia MV Marrani E Maccora I Pagnini I The off-label use of anakinra in pediatric systemic autoinflammatory diseases. Ther Adv Musculoskelet Dis. (2020) 12:1–19. 10.1177/1759720X20959575 PMC7580132 33149772

[4] Mehta P Cron RQ Hartwell J Manson JJ Tattersall RS. Silencing the cytokine storm: the use of intravenous anakinra in haemophagocytic lymphohistiocytosis or macrophage activation syndrome. Lancet Rheumatol. (2020) 2(6):e358–67. 10.1016/S2665-9913(20)30096-5 32373790 PMC7198216

[5] De Rose DU Campi F Maddaloni C Ronci S Caoci S Savarese I Off-label use of anakinra in inflammatory conditions in neonates and infants up to 3 months of age: a case series and a review of the literature. Paediatr Drugs. (2025) 27(3):293–305. 10.1007/s40272-024-00679-x 39804459 PMC12031743

[6] Green EA Metz D Galinsky R Atkinson R Skuza EM Clark M Anakinra Pilot—a clinical trial to demonstrate safety, feasibility and pharmacokinetics of interleukin 1 receptor antagonist in preterm infants. Front Immunol. (2022) 13:1–14. 10.3389/fimmu.2022.1022104 PMC9647081 36389766

[7] Engeroff P Belbézier A Monsel A Klatzmann D. Anakinra reduces lung inflammation in experimental acute lung injury. Immunity Inflamm Dis. (2022) 10(2):123–9. 10.1002/iid3.548 PMC8767508 34889061

[8] Eldredge LC Creasy RS Presnell S Debley JS Juul SE Mayock DE Infants with evolving bronchopulmonary dysplasia demonstrate monocyte-specific expression of IL-1 in tracheal aspirates. Am J Physiol Lung Cell Mol Physiol. (2019) 317(1):L49–56. 10.1152/ajplung.00060.2019 30969811

[9] Green EA Garrick SP Peterson B Berger PJ Galinsky R Hunt RW The role of the interleukin-1 family in complications of prematurity. Int J Mol Sci. (2023) 24(3):2795. 10.3390/ijms24032795 36769133 PMC9918069

[10] Humberg A Fortmann I Siller B Kopp MV Herting E Göpel W Preterm birth and sustained inflammation: consequences for the neonate. Semin Immunopathol. (2020) 42(4):451–68. 10.1007/s00281-020-00803-2 32661735 PMC7508934

[11] Li Q Li C Rao Y Han D Wang X Yang L Dynamic association between plasma interleukin-1 family concentrations and bronchopulmonary dysplasia in extremely premature infants. J Perinatol. (2026) 46:182–7. 10.1038/s41372-025-02275-4 40159578

[12] Nold MF Mangan NE Rudloff I Cho SX Shariatian N Samarasinghe TD Interleukin-1 receptor antagonist prevents murine bronchopulmonary dysplasia induced by perinatal inflammation and hyperoxia. Proc Natl Acad Sci U S A. (2013) 110(35):14384–9. 10.1073/pnas.1306859110 23946428 PMC3761642

[13] Bui CB Kolodziej M Lamanna E Elgass K Sehgal A Rudloff I Interleukin-1 receptor antagonist protects newborn mice against pulmonary hypertension. Front Immunol. (2019) 10:1–15. 10.3389/fimmu.2019.01480 31354700 PMC6637286

[14] Durlak W Thébaud B. BPD: latest strategies of prevention and treatment. Neonatology. (2024) 121(5):596–607. 10.1159/000540002 39053447

[15] Neven B Marvillet I Terrada C Ferster A Boddaert N Couloignier V Long-term efficacy of the interleukin-1 receptor antagonist anakinra in ten patients with neonatal-onset multisystem inflammatory disease/chronic infantile neurologic, cutaneous, articular syndrome. Arthritis Rheum. (2010) 62(1):258–67. 10.1002/art.25057 20039428

[16] Diorio C Vatsayan A Talleur AC Annesley C Jaroscak JJ Shalabi H Anakinra utilization in refractory pediatric CAR T-cell associated toxicities. Blood Adv. (2022) 6(11):3398–403. 10.1182/bloodadvances.2022006983 35395068 PMC9198909

[17] Fingerhutová Š Jančová E Doležalová P. Anakinra in paediatric rheumatology and periodic fever clinics: is the higher dose safe? Front Pediatr. (2022) 10:823847. 10.3389/fped.2022.823847 35321008 PMC8936593

[18] Kelly SB Sébire H Brochu ME Briota S Sarret P Sébire G. Interleukin-1: an important target for perinatal neuroprotection? Neural Regen Res. (2023) 18(1):47–50. 10.4103/1673-5374.341044 35799507 PMC9241389

